# FoxO3 Activation Alleviates Doxorubicin‐Induced Cardiomyopathy by Enhancing Autophagic Flux and Suppressing mTOR/ROS Signalling

**DOI:** 10.1111/jcmm.70775

**Published:** 2025-08-10

**Authors:** Zao‐Shang Chang, Le Wang, Ju‐Xiang Zhou, Mei‐Xiu‐Li Li, Meng‐Yun Yang, Bin Luo, Jia‐Jun Liu, Xiao‐Ye Sun, Jing‐Bo Xia

**Affiliations:** ^1^ Department of Physiology, Pu ai Medical School Shaoyang University Shaoyang Hunan China; ^2^ Key Laboratory of Regenerative Medicine, Ministry of Education Jinan University Guangzhou Guangdong China; ^3^ Department of Pharmacy The Central Hospital of Shaoyang Shaoyang Hunan China; ^4^ School of Nursing Shaoyang University Shaoyang Hunan China; ^5^ Guangdong Provincial Key Laboratory of Physical Activity and Health Promotion Guangzhou Sport University Guangzhou Guangdong China

**Keywords:** autophagy, cardiomyopathy, doxorubicin, *FoxO3*, reactive oxygen species

## Abstract

Doxorubicin (DOX) is an effective chemotherapy drug, but its use is limited by cardiotoxicity, known as DOX‐induced cardiomyopathy. The transcription factor FoxO3, which regulates autophagy and oxidative stress, has unclear mechanisms in this condition. We found that DOX‐induced cardiomyopathy involved cardiac atrophy, cardiac dysfunction, fibrosis and mitochondrial damage. DOX reduced H9c2 cardiomyocyte viability and glutathione levels (GSH), increased reactive oxygen species (ROS), malondialdehyde (MDA) and lactate dehydrogenase (LDH) and inhibited superoxide dismutase 2 (*SOD2*) and catalase (*CAT*) expression. DOX also suppressed *FoxO3* activation and increased the autophagy protein LC3 II/I ratio. Overexpressing *FoxO3* enhanced LC3B, Beclin 1 and autophagic flux, while reducing p62 and suppressing mTOR activation in heart. Brefeldin A1 (BafA1), an autophagy inhibitor and rapamycin (Rapa), an autophagy activator, were administered to H9c2 cardiomyocytes to elucidate the regulatory mechanism of *FoxO3*. Mechanically, our data revealed that *FoxO3* overexpression enhanced autophagy and suppressed ROS production and mTOR activation in both in vitro and in vivo models of DOX exposure. Collectively, targeting *FoxO3* to enhance protective autophagy may offer a therapeutic strategy against DOX‐induced cardiomyopathy.

**Abbreviations**
AAVFoxO3: adeno‐associated virus of FoxO3
AAVNC: adeno‐associated virus of negative control
CO: cardiac output
EF: ejection fraction
FS: fractional shortening
IVDd: end diastolic ventricular septal thickness
IVSd: end systolic ventricular septal thickness
LV: left ventricular
LVAWd: left ventricular anterior wall thickness in diastole
LVAWs: left ventricular anterior wall thickness in systole
LVEDD: left ventricular end diastolic diameter
LVEDV: left ventricular end diastolic volume
LVESD: left ventricular end systolic diameter
LVESV: left ventricular end systolic volume
LVIDd: left ventricular internal diastolic dimension
LVISd: left ventricular internal systolic dimension
LVPWd: left ventricular posterior wall thickness in diastole
LVPWs: left ventricular posterior wall thickness in systole
OEFoxO3: overexpression plasmid of FoxO3
OENC: overexpression plasmid of negative control
SV: stroke volume

## 1 Introduction

The utilisation of anthracycline chemotherapy drugs has resulted in notable advancements in the survival rates of cancer patients. Nevertheless, their use leads to an escalation in mortality due to the adverse effects of treatment, particularly involving cardiovascular diseases [1]. A conducted prospective registry analysis revealed a substantial prevalence of 37.5% in therapy‐induced cardiotoxicity associated with anticancer treatments [2]. DOX is a highly effective chemotherapeutic agent that is frequently employed either as a monotherapy or in combination for the treatment of various cancers. However, its significant dose‐dependent cardiotoxicity can lead to irreversible heart failure in the clinic [3]. It has been reported that arrhythmia, myocarditis and heart failure have occurred in tumour patients treated with DOX [4]. Previous publications indicate that the pathogenesis of DOX‐induced cardiomyopathy incorporates various factors, including apoptosis of cardiomyocytes, production of ROS and mitochondrial dysfunction [5, 6, 7]. To date, there are few effective treatment options with a relatively poor prognosis for DOX cardiotoxicity. Therefore, uncovering the underlying mechanism and exploring potential preventive strategies is urgently needed.

Oxidative stress, characterised by an imbalance between the excessive generation of ROS in cells and tissues and the antioxidant defence mechanisms, plays a central role in the pathogenesis of doxorubicin‐induced cardiomyopathy [8]. *SOD2* and *CAT* are prominent antioxidants in cardiomyocytes during normal physiological circumstances, and research indicates that these antioxidant enzymes participate in preserving cellular ROS homeostasis in doxorubicin‐induced cardiotoxicity [9]. Moreover, accumulating evidence has depicted mitochondrial dysfunction as the involvement of adverse cardiac remodelling in the development of DOX cardiomyopathy [10]. Mitochondrial dynamics and biosynthesis provide indispensable roles in maintaining cellular energy metabolism and cardiac function, and a deficiency in mitochondrial oxidative metabolism results in contraction defects and heart failure in patients and experimental animals [11, 12]. Consequently, ROS and mitochondrial bioenergetics are attracting extensive attention as important therapeutic targets for DOX‐induced cardiomyopathy.

Conversely, autophagy, a highly conserved process, facilitates the degradation of cellular constituents, such as malfunctioning organelles and aggregated proteins, thereby mitigating oxidative damage and reducing ROS levels [13]. Recent research has shown that *atg7*‐based autophagy activation effectively reverses the decline in cardiac function in DOX‐induced cardiotoxicity [14]. At the clinical level, autophagy‐related genes provide protection from DOX cardiotoxicity in patient's cardiomyocytes [15]. Furthermore, evidence has been reported that autophagy attenuates phenylephrine‐induced cardiac hypertrophy by inhibiting protein kinase B (Akt)/mammalian target of rapamycin (mTOR) signalling [16]. Therefore, the regulation of autophagy might be a target of treatment for DOX‐induced cardiomyopathy. mTOR signalling, one of the major regulators of cellular metabolism, is key to cellular growth and proliferation [17]. mTOR protein kinases exist in two different core complexes, mTOR complex I and II (mTORC1 and mTORC2, respectively), differ in their regulation and function and sensitivity to rapamycin [18]. Previous work has indicated the effect of DOX involving inhibition of mTOR, which may be related to DOX destroying the balance of energy metabolism in myocardial cells to elevate cardiomyocyte apoptosis [19]. Apparently, DOX is able to modulate mTOR targets by affecting intracellular energy metabolism and oxidative stress in cardiomyocytes.

The FoxO transcription factors are members of the FOX genes that share a common DNA domain (called the Forkhead domain). The FoxO family consists of four members, namely *FoxO1*, *FoxO3*, *FoxO4* and *FoxO6* [20]. The FoxO3 transcription factor, belonging to the FoxO subclass, is a critical regulator of diverse cellular processes, including cell cycle, apoptosis, differentiation, stress resistance and autophagy [21]. Recent research has demonstrated that *FoxO3* regulates cardiomyocyte cell size associated with autophagy [22]. Indeed, *FoxO3* was shown to transcriptionally control autophagy by regulating *Bnip*, *ATG* genes and *Gabarapl1* in cultured cardiomyocytes and in vivo [23, 24, 25]. While numerous studies have clarified the function of *FoxO3*, its roles in DOX‐induced cardiomyopathy as mediated by *FoxO3* remain unclear, and many mechanisms have yet to be identified.

To this end, our current study is designed to explore the role of *FoxO3* in the pathogenesis of DOX cardiomyopathy and the underlying mechanisms. Collectively, taking advantage of the overexpression of *FoxO3* in H9c2 cardiomyocytes and the heart, *FoxO3* is found to protect against DOX cardiotoxicity by activating autophagy, inhibiting intracellular ROS and mTOR signalling, thus revealing the therapeutic potential of *FoxO3* as a target in DOX cardiomyopathy clinically.

## 2 Materials and Methods

See [Link], [Link].

## 3 Results

### 3.1 DOX Administration Induced Cardiac Atrophy and Dysfunction

**FIGURE 1:**
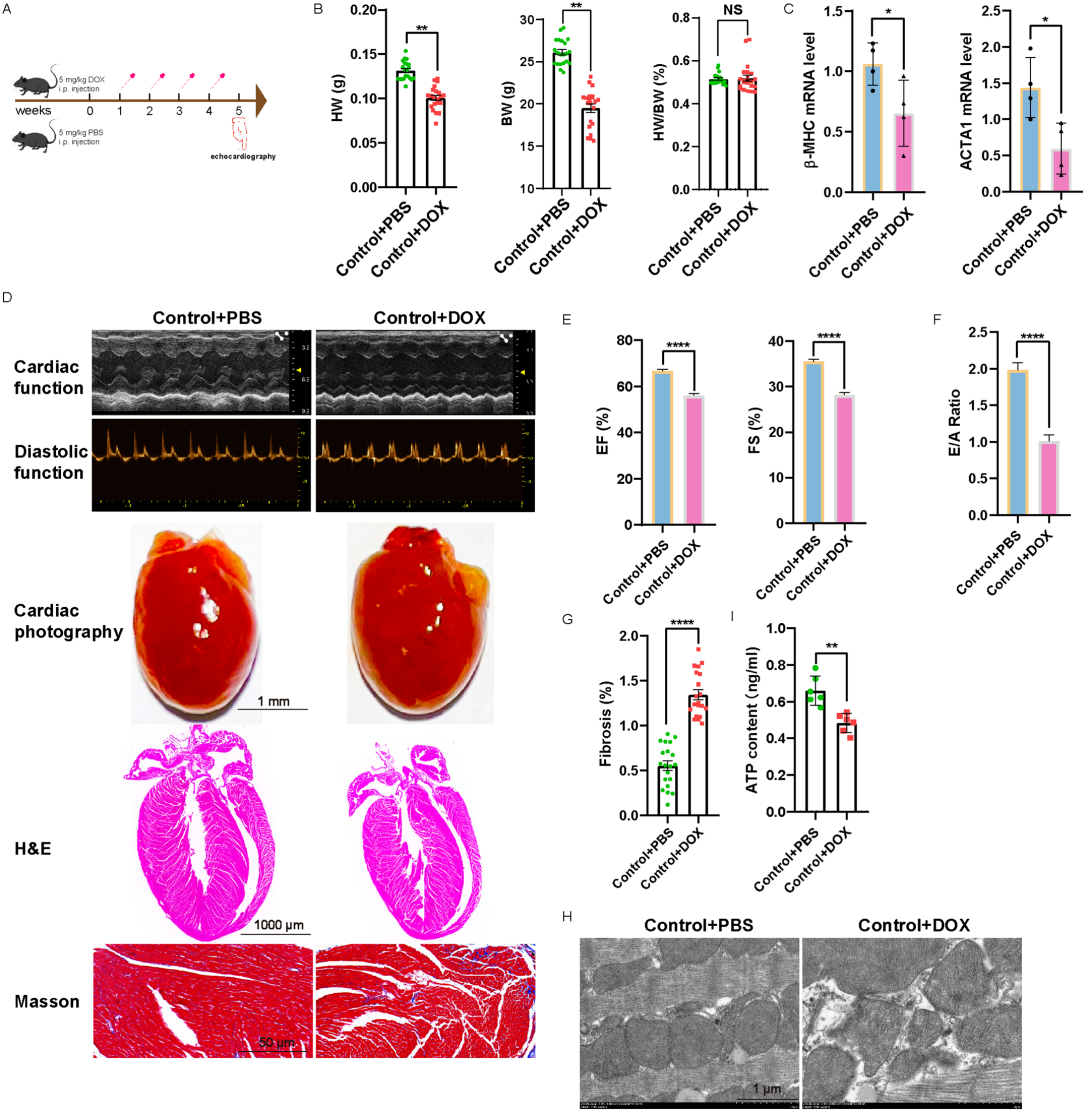
DOX induces cardiac atrophy and cardiac dysfunction in vivo. (A) Schematic diagram depicting the protocol for DOX and PBS intraperitoneal injection; PBS was used as negative control. The figure was drawn by Figdraw. (B) Changes in heart weight (HW), body weight (BW) and the ratio of HW/BW (*n* = 21 for each group). (C) *β‐MHC* and *ACTA1* were analysed by qRT‐PCR (*n* = 4 hearts for each group). (D) Cardiac function was evaluated by echocardiography (top panel). Representative images of cardiac photography, H&E staining and Masson staining. (E) The ejection fractions (EF) and fraction shortening (FS) were calculated by M‐mode echocardiography (*n* = 15 for each group). (F) The E/A ratio was calculated (*n* = 15 for each group). (G) Cardiac fibrosis was quantified after Masson trichrome staining (*n* = 20 hearts per group). (H) Transmission electron microscopy (TEM) images of heart tissues from each group (*n* = 3 for each group). (I) ATP content in myocardial tissue was quantified (*n* = 6 for each group). Data are presented as mean ± SEM, *p < 0.05, **p < 0.01 and ****p < 0.0001.

In order to assess the impact of cardiac damage in vivo, we administered intraperitoneal injections of DOX to C57BL/6J mice, while the Control group received an equivalent amount of PBS over a period of 4 weeks (Figure 1A). In line with clinical observations of weight loss in patients undergoing DOX treatment, we noted a substantial decrease in body weight (BW) among the DOX‐treated mice (Figure 1B). Additionally, the DOX treatment resulted in cardiac atrophy, as indicated by a significant reduction in overall heart weight (HW) (Figure 1B). However, the ratios of heart to body weight (HW/BW), as depicted in Figure 1B, did not exhibit any significant differences. Furthermore, the induction of cardiac atrophy by DOX was observed, as determined by RT‐PCR detection, along with a suppression of markers for cardiac hypertrophy, namely β‐myosin heavy chain (*β‐MHC*) and actin α1 (*ACTA1*) mRNA expression (Figure 1C). Our study indicated that DOX treatment induced cardiac atrophy.

To investigate the impact of DOX‐induced cardiac cardiotoxicity, we assessed cardiac function using echocardiography. Our findings revealed that DOX administration resulted in an accelerated decline in cardiac function and diastolic function, as indicated by a decrease in ejection fraction (EF), fractional shortening (FS) and the E/A ratio and an increase in left ventricular end‐systolic diameter (LVESD) and left ventricular end‐systolic volume (LVESV) (Figure 1D–F and Figure S1A). Moreover, significant differences were observed in left ventricular posterior wall thickness (LVPW), left ventricular anterior wall thickness (LVAW), left ventricular mass (LV Mass), stroke volume (SV) and cardiac output (CO) Figure S1A. Subsequently, the examination of cardiac remodelling and fibrosis was conducted using H&E and Masson staining techniques. Our analysis revealed that hearts from the DOX group exhibited evident myofiber disarray as observed in the H&E staining, along with interstitial fibrosis in the myocardial tissues, in comparison to the Control group (Figure 1D,G). Mitochondrial dysfunction is a well‐documented contributor to the pathogenesis of DOX‐induced cardiomyopathy [26]. Hence, the transmission electron microscopy results showed that the normal myocardial mitochondria presented a normal and complete morphology, but swelling, vacuolisation, disorganised and decreased cristae density, and an incomplete structure were observed in the DOX‐treated myocardial interfibrillar mitochondria (Figure 1H). To determine the effect of DOX on cardiomyocyte energy, myocardial adenosine triphosphate (ATP) levels were measured. As shown in Figure 1I, ATP levels in the cardiac tissue of mice in the DOX group were lower than those in the Control group. Collectively, these results illustrated that DOX treatment induced a cardiomyopathy phenotype, manifested cardiac dysfunction, fibrosis, cardiac remodelling and mitochondrial damage.

### 3.2 FoxO3 Transcriptional Activity Was Inhibited by DOX Treatment In Vivo and In Vitro

**FIGURE 2:**
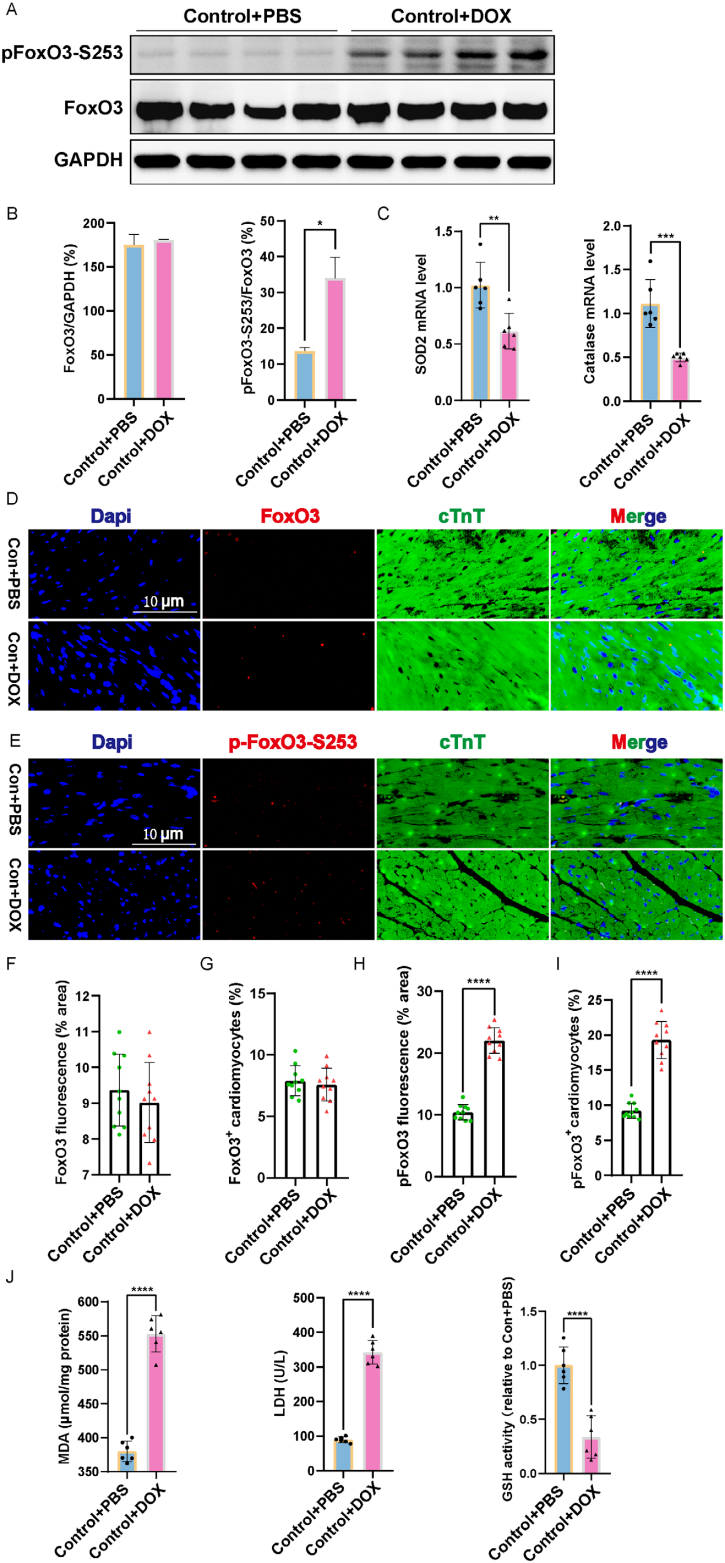
FoxO3 transcriptional activity and the expression of antioxidant genes are suppressed in DOX cardiomyopathy. (A) Representative images of western blot for FoxO3 and phospho‐FoxO3‐Ser253. (B) FoxO3 expression and phospho‐FoxO3/total FoxO3 ratio (*n* = 4). (C) *SOD2* and *CAT* mRNA expression (*n* = 6). (D‐E) FoxO3 and p‐FoxO3‐S253 expression were detected by immunofluorescence staining. (F‐G) Quantification of FoxO3 fluorescence intensity and the FoxO3^+^ cTnT^+^ cells (*n* = 10 per group). (H‐I) Quantification of p‐FoxO3‐S253 fluorescence intensity and the p‐FoxO3‐S253^+^ cTnT^+^ cells (*n* = 10 per group). (J) MDA, LDH and GSH enzyme activity in cardiac tissue were quantified (*n* = 6 per group). Data are presented as Mean ± SEM, *p < 0.05, **p < 0.01, ***p < 0.001 and ****p < 0.0001.

To explore the potential association between *FoxO3* and pathological cardiomyopathy, the expression of FoxO3 in mouse myocardial tissue was quantified through western blot analysis. The phosphorylation of FoxO3 by Akt leads to its association with 14‐3‐3 proteins and subsequent nuclear rejection, retention and degradation in the cytoplasm, thus inactivating the FoxO3 pathway [27]. Notably, the phosphorylation of FoxO3 at Ser 253 was observed to be significantly increased following stimulation with DOX, indicating that DOX inhibits the transcriptional activity of FoxO3 (Figure 2A,B). However, the total FoxO3 protein remained unchanged in myocardial tissue treated with DOX (Figure 2A,B). Besides, the in vivo expression and localisation of FoxO3 in cardiomyocytes were measured by immunofluorescence staining; we found that hearts after DOX treatment showed no difference in both the mean fluorescence intensity (MFI) of FoxO3 and the percentage of FoxO3^+^ cTNT^+^ cells (Figure 2D,F,G). Meanwhile, DOX promoted FoxO3 phosphorylation in the nucleus, significantly increasing both the MFI of pFoxO3 and the percentage of pFoxO3^+^ cTNT^+^ cells (Figure 2E,H,I). Given that FoxO3 is a vital transcription factor that promotes the transcription of antioxidant genes such as *SOD2* and *CAT* [28], we noted a significant decrease in the mRNA levels of *SOD2* and *CAT* in myocardial tissues treated with DOX compared to the WT + PBS group (Figure 2C). It has been reported that oxidative stress participates in the injury of cardiomyocytes induced by DOX [29]. Next, we found that the treatment of DOX significantly increased the contents of myocardial injury markers, MDA and LDH, in mice myocardial tissue, and dramatically reduced GSH levels versus the Control group (Figure 2J). Taken together, these data indicated that the FoxO3 signalling pathway was related to DOX‐induced cardiomyopathy in mice.

Consequently, we further elucidate potential mechanisms underlying the protective effects of *FoxO3* against DOX‐induced damage in H9c2 cardiomyocytes. In order to determine the most suitable dosage range of DOX for H9c2 cardiomyocytes, we conducted CCK8 assays to assess cell viability. H9c2 cardiomyocytes were incubated with varying concentrations of DOX (0, 0.5, 1 and 2 μM) for a duration of 24 h. The results depicted in Figure S1A demonstrate a significant concentration‐dependent suppression of H9c2 cell viability upon exposure to DOX. Then, we selected a dosage of 0.5 μM DOX as the optimal concentration for subsequent investigations in H9c2 cardiomyocytes, and the Control group was supplemented with PBS. In order to measure the levels of ROS, fluorescent probes CM‐H_2_DCFDA were utilised in conjunction with flow cytometry. Our findings indicated that DOX significantly increased the mean fluorescence intensity (MFI) in the cardiomyocyte group (Figure S2B,C). Obviously, the content of MDA was increased, and the expression of *SOD2* and *CAT* mRNA was diminished following DOX treatment (Figure S2D,G). Consistent with the in vivo results, we observed a significant increment of the ratio of pFoxO3‐Ser253 to FoxO3 in H9c2 cardiomyocytes exposed to DOX, compared to those treated with PBS alone (Figure S2E,F). This demonstrated that DOX exposure induced oxidative stress and inhibited the activation of *FoxO3* in H9c2 cardiomyocytes.

### 3.3 Autophagy Mediated DOX‐Induced Cardiomyopathy

**FIGURE 3:**
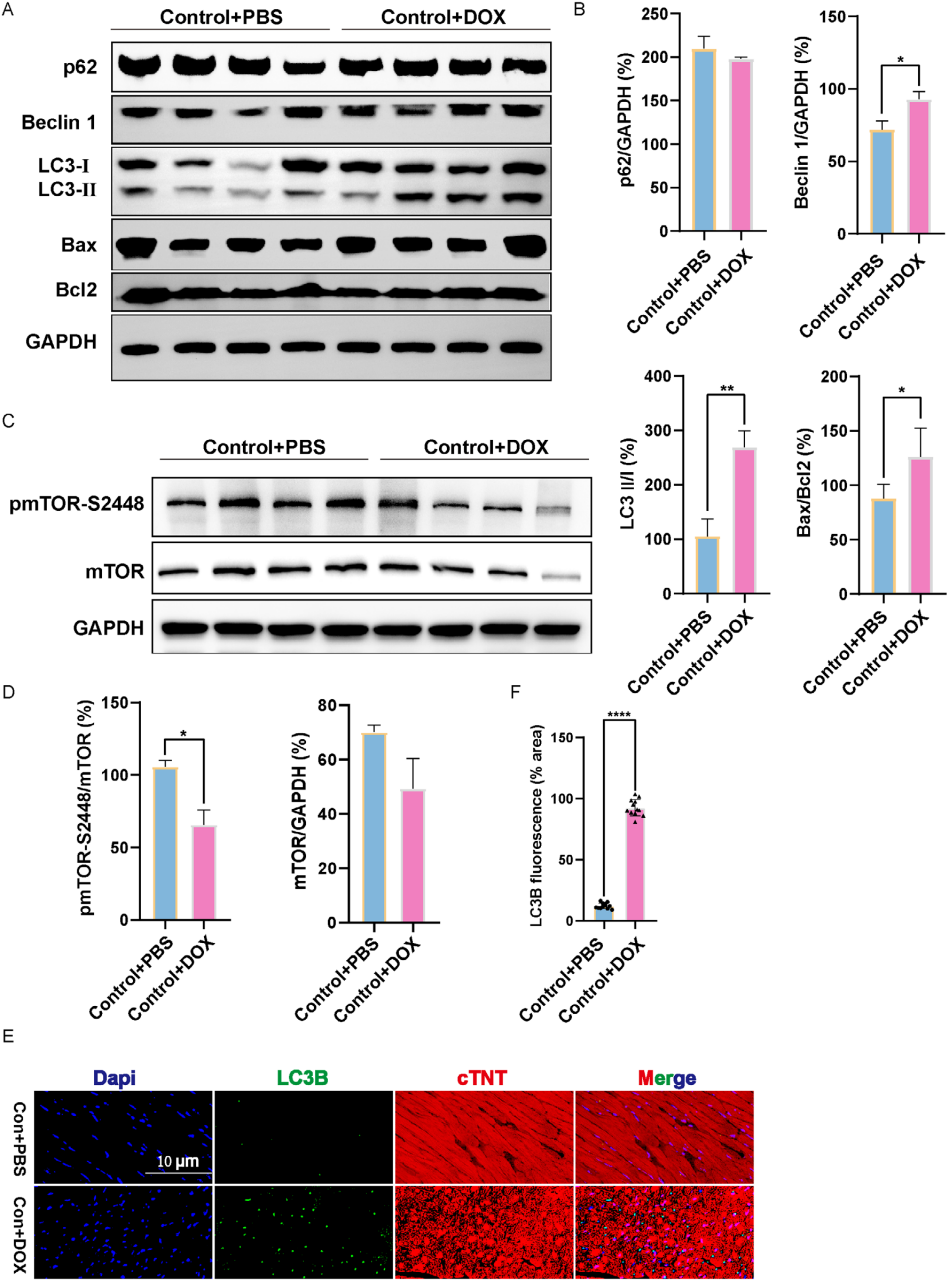
Effects of DOX on autophagy and mTOR in heart tissue. (A) Western blot images of p62, Beclin 1, LC3 II/I, Bax and Bcl2. (B) The relative expression levels of target proteins were quantified as the percentage of Control group (*n* = 4). (C) Western blot images of mTOR and p‐mTOR‐Ser2448. (D) The relative expression levels of target proteins were quantified as the percentage of Control (*n* = 4). (E) The localisation and expression of LC3B in myocardium were detected by immunofluorescence staining. Representative images of LC3B were shown (green). cTnT, cardiomyocytes marker. (F) Quantification of LC3B fluorescence intensity was performed (*n* = 12 per group). Data are presented as Mean ± SEM, *p < 0.05, **p < 0.01 and ****p < 0.0001.

The study demonstrated that autophagy in the myocardium plays a crucial role in the regulation of various cardiovascular diseases, including DOX‐induced cardiotoxicity [30]. Previous studies have reported that several key autophagy proteins, including ATG5, ATG6/Beclin‐1, ATG7, ATG12 and p62, are in connection with inducing autophagy [31]. In order to investigate the potential efficacy of autophagy in the development of DOX‐induced cardiomyopathy, we conducted an examination of the protein expression levels of several autophagy‐related proteins, namely LC3B, Beclin 1 and p62, in the myocardium of mice treated with DOX. The level of MAP1LC3/LC3‐II is recognised as an acknowledged marker of autophagic activity [32]. The results obtained from western blot analysis indicated a significant increase in the protein expression of the LC3 II/I ratio in the DOX group compared to the Control group (Figure 3A,B). A decrease in the abundance of p62 is considered indicative of enhanced autophagic flux, and Beclin‐1 associates with a preautophagosomal complex, which is required for the localisation of autophagic proteins to the preautophagosomal structure [33, 34]. We found that the protein expression level of Beclin 1 was higher in DOX mice than in the Control group (Figure 3A,B). However, subsequent experiments revealed no statistically significant disparity in the protein expression levels of p62 in the myocardium between mice subjected to DOX treatment and those administered PBS (Figure 3A,B). Autophagy, a multifaceted process, can be modulated by diverse signalling pathways, for example, mTOR, one of the hallmarks of cellular metabolism. Thus, we assessed the protein expressions of p‐mTOR‐Ser2448 and mTOR in myocardial tissue treated with DOX. Remarkably, we observed noteworthy reductions in the ratio of p‐mTOR/mTOR in DOX‐induced cardiomyopathy mice compared to those in the Control group (Figure 3C,D), confirming the mTOR signalling pathway's role in regulating DOX‐induced cardiomyopathy. Besides, as shown in Figure 3E,F, the in vivo expression and localisation of LC3B in cardiomyocytes were measured by immunofluorescence staining, and the expression of LC3B was dramatically increased by DOX compared to the PBS‐treated mice. Thus, DOX further increased apoptosis with upregulation of Bax/Bcl2 protein expression subjected to DOX treatment (Figure 3A,B). Our in vivo studies suggest that the cardioprotective effect of *FoxO3* may lie in the activation of autophagy and depend on the mTOR pathway in DOX‐induced cardiomyopathy.

### 3.4 Autophagy Mediated DOX‐Induced H9c2 Cardiomyocytes Oxidative Stress In Vitro

**FIGURE 4:**
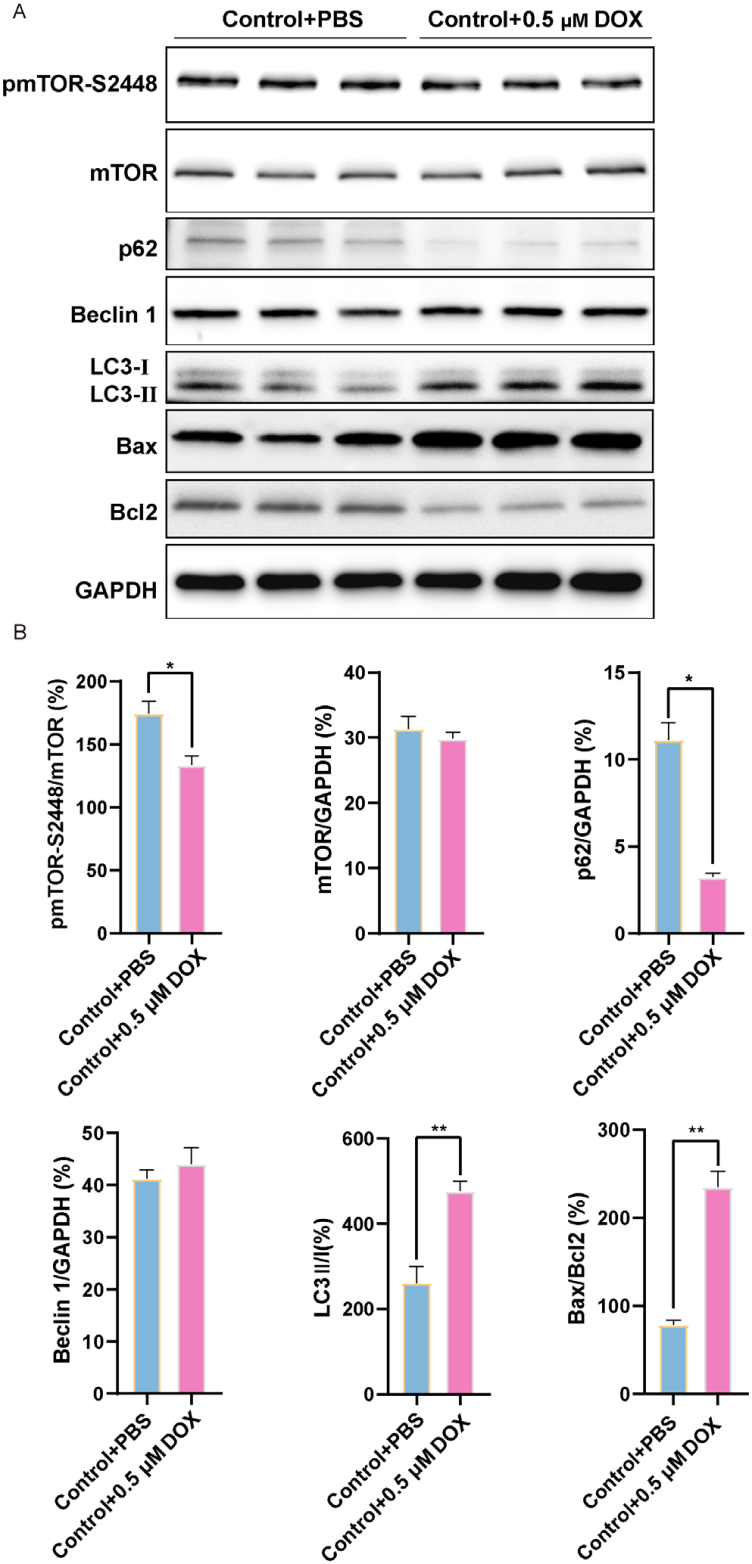
Effects of DOX on autophagy, mTOR and apoptosis in H9c2 cardiomyocytes. (A) Western blot images of p‐mTOR‐Ser2448, mTOR, p62, Beclin 1, LC3 II/I, Bax and Bcl2 in H9c2 cardiomyocytes. (B) The relative expression levels of target proteins were quantified as the percentage of the Control group (*n* = 3). Data are presented as Mean ± SEM, *p < 0.05 and **p < 0.01.

Subsequently, we tested the autophagy‐associated protein levels of LC3B, p62 and Beclin 1 after the H9c2 cardiomyocytes were treated with DOX. DOX significantly promoted the conversion of LC3 I to LC3 II, which was consistent with those obtained at the tissue samples, and downregulated the p62 levels accordingly (Figure 4A,B). However, there was no significant change in Beclin 1 expression in DOX‐induced H9c2 cardiomyocytes (Figure 4A,B). As shown in Figure 4A,B, we found that the changes in the ratio of p‐mTOR/mTOR were decreased after DOX stimulation of H9c2 cardiomyocytes in contrast to those in the Control group, consistent with in vivo experiments. Moreover, compared with the Control group, DOX treatment promoted the apoptosis of H9c2 cardiomyocytes (Figure 4A,B). Briefly, these results established a critical role for autophagy and mTOR signalling in mediating DOX‐induced H9c2 cardiomyocytes oxidative stress.

### 3.5 *FoxO3* Overexpression Protected Against DOX‐Induced ROS by Activating Autophagy

**FIGURE 5:**
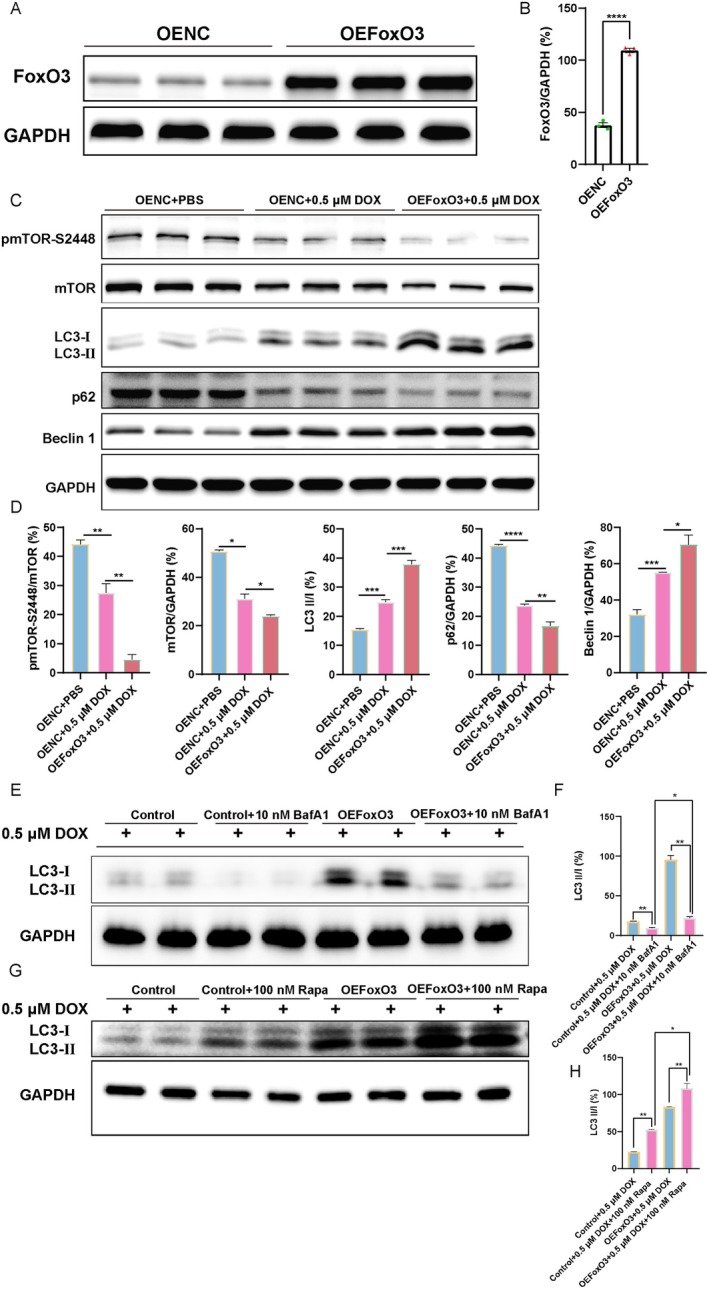
Overexpression of *FoxO3* increases autophagy and inhibits activation of mTOR in H9c2 cardiomyocytes. (A‐B) Validation of FoxO3 overexpression (*n* = 3). (C) Western blot images of p‐mTOR‐Ser2448, mTOR, p62, Beclin 1 and LC3 II/I. (D) The relative expression levels of target proteins were quantified as the percentage of Control (*n* = 3). (E) Western blot images of LC3 II/I. (F) The relative expression levels of target proteins were quantified as the percentage of Control group. (G) Western blot images of LC3 II/I. (H) The relative expression levels of target proteins were quantified as the percentage of Control group. Data are presented as Mean ± SEM, *p < 0.05, **p < 0.01, ***p < 0.001 and ****p < 0.0001.

To further elucidate the mechanism of *FoxO3* in DOX‐induced cardiomyopathy, *FoxO3* overexpression was achieved by transfecting a murine *FoxO3* expression plasmid into H9c2 cells. The successful verification of *FoxO3* overexpression was confirmed through western blot analysis, as depicted in Figure 5A,B. Subsequently, the administration of DOX significantly augmented the expression of LC3 II/I and Beclin 1 in the OENC+DOX group, and this effect was further potentiated by the overexpression of *FoxO3* (Figure 5C,D). Conversely, DOX exposure could suppress the expression of p62, which was significantly decreased by *FoxO3* overexpression in H9c2 cardiomyocytes treated with DOX (Figure 5C,D). Additionally, we discovered that the DOX‐induced downregulation of the p‐mTOR/mTOR ratio in the OENC+DOX group was further enhanced due to *FoxO3* overexpression (Figure 5C,D). Meanwhile, Brefeldin A1, an autophagy inhibitor, was administered to H9c2 cardiomyocytes to elucidate the regulatory mechanism of *FoxO3*. A significant reduction of LC3‐II/I protein ratio was demonstrated in the Control+BafA1 group compared with the Control group (Figure 5E,F). However, LC3‐II/I protein expression was significantly upregulated in the OEFoxO3+ BafA1 group compared with the Control+BafA1 group (Figure 5E,F). Furthermore, the effects of FoxO3 overexpression on LC3B were significantly promoted by the classic autophagy activator Rapa, which works with the inhibition of mTOR activity (Figure 5G,H). Collectively, overexpression of *FoxO3* increased LC3 II/I levels after blocking and activating the autophagic flow using BafA1 and Rapa in H9c2 cardiomyocytes.

**FIGURE 6:**
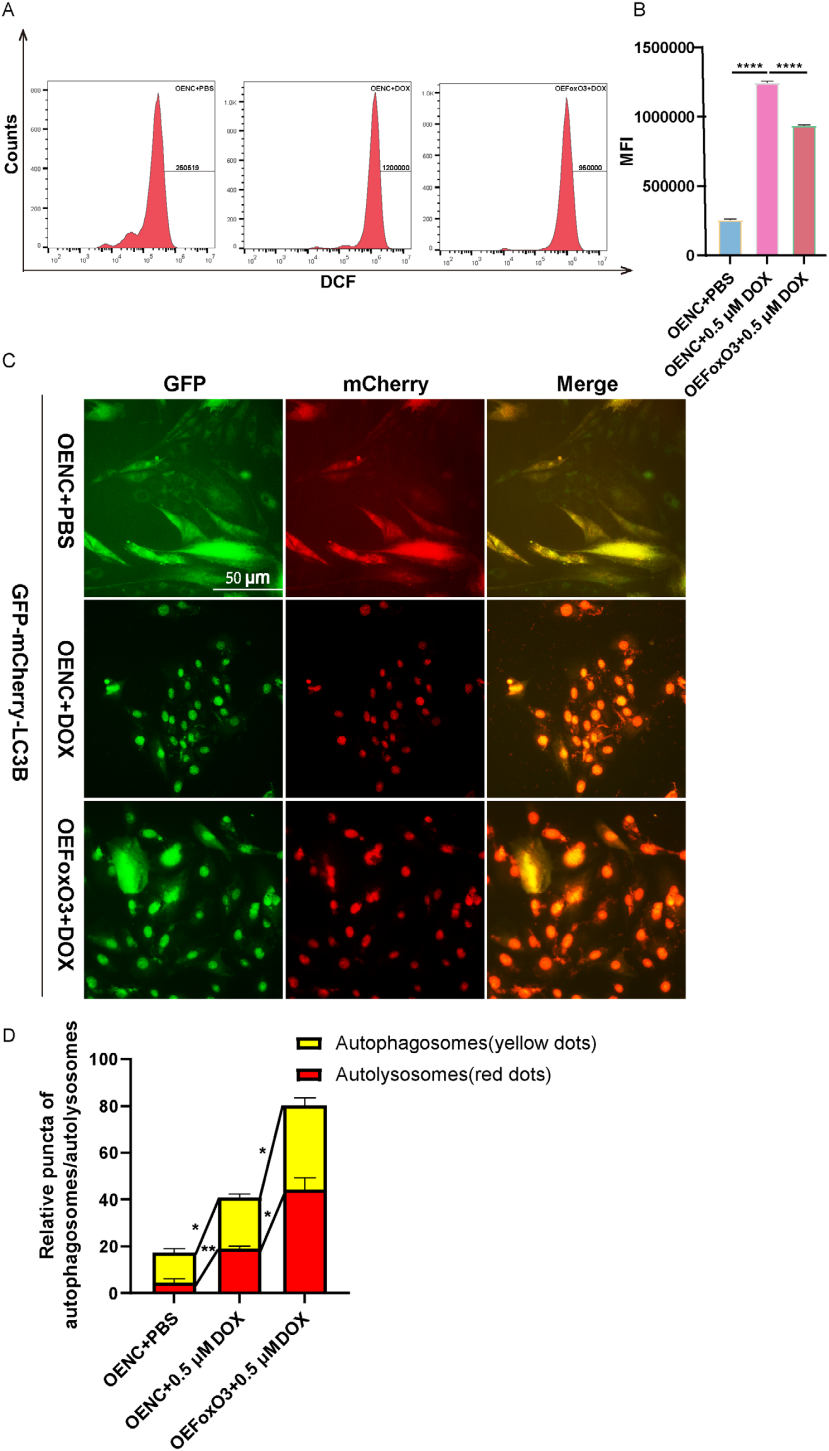
Overexpression of *FoxO3* inhibits intracellular ROS and enhances autophagy flow. (A) ROS detection by CM‐H_2_DCFDA in flow cytometry. (B) Quantification of mean fluorescence intensity (MFI) (*n* = 3). (C‐D) mCherry‐red fluorescent protein‐green fluorescent protein (GFP)‐LC3B adenovirus was transfected into H9c2 cardiomyocytes to observe changes in autophagic flux. The yellow puncta and red puncta represent autophagosomes and autolysosomes, respectively, which were counted on 30 cells (*n* = 3 for each group). Data are presented as Mean ± SEM, *p < 0.05, **p < 0.01 and ****p < 0.0001.

Besides, as depicted in Figure 6A,B, the treatment of DOX significantly augmented the MFI of DCF^+^ cells in the OENC+DOX group, which was subsequently suppressed by the overexpression of *FoxO3*. We then transduced H9c2 cardiomyocytes with GFP‐mCherry‐LC3B adenovirus and analysed the changes in autophagy by counting the number of red and yellow puncta (Figure 6C). Simultaneously, we observed that a few punctate fluorescent patterns in the untreated Control cells identified the autophagosomes (yellow dots) and autolysosomes (red dots), whereas doxorubicin‐treated H9c2 cardiomyocytes exhibited a striking accumulation of punctate fluorescent patterns, which was boosted by the remarkable accumulation of punctate fluorescent patterns in *FoxO3* overexpressed H9c2 cardiomyocytes (Figure 6C,D). Our findings proved that the forced expression of *FoxO3* can strengthen the progression of autophagy flow and alleviate the accumulation of DOX‐induced ROS in H9c2 cardiomyocytes.

### 3.6 *FoxO3* Overexpression Protected Against DOX‐Induced Cardiomyopathy by Activating the Autophagy

**FIGURE 7:**
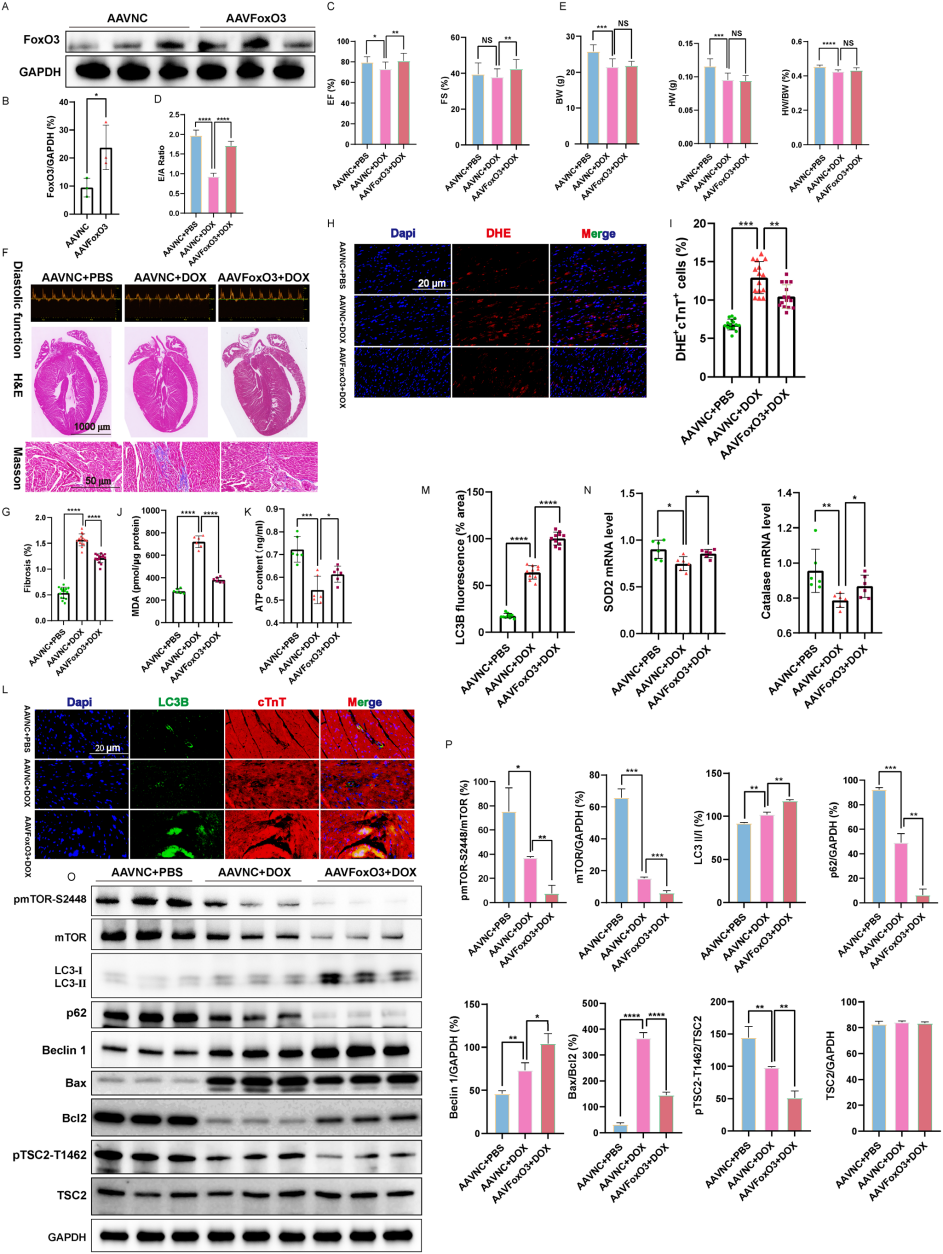
Overexpression of *FoxO3* protects DOX‐induced cardiomyopathy by activating autophagy. (A‐B) Validation of FoxO3 overexpression in hearts (*n* = 3). (C) The ejection fractions (EF) and fraction shortening (FS) were calculated by M‐mode echocardiography (*n* = 20 for each group). (D) Diastolic function (E/A ratio) was calculated (*n* = 20 for each group). (E) Changes of heart weight (HW), body weight (BW) and the ratio of HW/BW in the AAVNC+PBS, AAVNC+DOX and AAVFoxO3 + DOX groups (*n* = 15 for each group). (F) At the end of the experiment, diastolic function was tested (top panel). Representative images of H&E staining and Masson staining. (G) Quantitative analysis of myocardial fibrosis (*n* = 15 for per group). (H) Representative images of dihydroethidium (DHE) staining (red). (I) Quantification of DHE^+^ cTnT^+^ cells in hearts (*n* = 15 for each group). (J) Quantification of MDA concentration in hearts (*n* = 6 hearts for each group). (K) Quantification of ATP productions in hearts (*n* = 6 hearts for each group). (L) The localisation and expression of LC3B in myocardium were detected by immunofluorescence staining. Representative images of LC3B were shown (green). cTnT, cardiomyocytes marker (*n* = 10 for each group). (M) Quantification of LC3B fluorescence intensity was performed. (N) Cardiac *SOD2* and *CAT* mRNA expression (*n* = 6). (O) Western blot images of p‐mTOR‐Ser2448, mTOR, p62, Beclin 1, LC3 II/I, Bax, Bcl2, p‐TSC2‐Thr1462 and TSC2. (P) The relative expression levels of target proteins were quantified (*n* = 3). Data are presented as Mean ± SEM, *p < 0.05, **p < 0.01, ***p < 0.001 and ****p < 0.0001.

Subsequently, to further discover the mechanism of *FoxO3* in DOX‐induced cardiomyopathy, we next constructed an adenoviral *FoxO3* overexpression system (AAV9‐OEFoxO3) to achieve heart‐specific *FoxO3* overexpression. We examined whether overexpression of *FoxO3* could protect against DOX‐induced cardiomyopathy by activating autophagy in vivo; the experimental scheme is shown in Figure S3A. As shown in Figure 7A,B, overexpression of *FoxO3* was confirmed by Western blot. The persistent EGFP expression of AAV9‐OEFoxO3 in mice after virus injection for 4 weeks was detected using a fluorescence microscope (Figure S3B). As expected, cardiac function and diastolic function were significantly increased by *FoxO3* overexpression in DOX‐treated mice (Figure 7C,D,F); other indicators related to cardiac function testing were shown in Figure S3C. The DOX‐induced ventricular remodelling was suppressed by overexpression of FoxO3, which was demonstrated by increased HW/BW ratio, H&E staining and decreased fibrosis (Figure 7E–G). Moreover, forced expression of *FoxO3* suppressed the DOX‐induced oxidative stress in hearts, which was proved by decreases in DHE^+^ cTnT^+^ cell number (Figure 7H,I), MDA production (Figure 7J) and increased ATP production (Figure 7K). Moreover, expression of *SOD2* and *CAT* was increased due to forced activation of FoxO3 (Figure 7N). Besides, *FoxO3* overexpression in DOX‐treated mice significantly promoted the expression of LC3B (Figure 7L,M). In addition, *FoxO3* overexpression protected against DOX‐induced cardiomyopathy by activating the autophagy in vivo, which was demonstrated by increased expression of LC3 II/I ratio, Beclin 1 and decreased p62 and p‐mTOR/mTOR ratio, as well as reducing the expression of apoptotic protein Bax/Bcl2 ratio (Figure 7O,P). TSC2, as a key negative regulator of mTOR [35], we observed less phosphorylation at the AKT‐dependent phosphorylation of TSC2 on Thr 1462 in the AAVNC+DOX group, while overexpression of *FoxO3* significantly inhibited the phosphorylation of TSC2 exposed to doxorubicin (Figure 7O,P). Altogether, these results further demonstrated that *FoxO3* protected against DOX‐induced cardiomyopathy through increasing autophagy and suppressing ROS/mTOR activity.

## 4 Discussion

In the present study, we revealed a previously uncovered role of *FoxO3* in doxorubicin‐induced cardiomyopathy. We found that the phosphorylation level of *FoxO3* expression was markedly increased in DOX‐treated hearts and H9c2 cardiomyocytes. Besides, *FoxO3* modulated DOX cardiomyopathy associated with the autophagy‐related gene *LC3B*, *p62* and *Beclin 1*, as well as mTOR signalling in vivo and in vitro. Furthermore, overexpression of *FoxO3* can strengthen autophagy and inhibit DOX‐induced ROS generation in H9c2 cardiomyocytes and heart. Mechanistically, we demonstrated that *FoxO3* protected against doxorubicin‐induced cardiomyopathy by activating autophagy in vivo and in vitro. These findings contribute to the advancement of cardio‐oncology and strengthen our understanding of the potential molecular mechanisms underlying DOX cardiomyopathy.

Cardiomyocyte death and adverse cardiac remodelling, which ultimately progress into heart failure, are common complications caused by anti‐cancer DOX chemotherapy [36]. According to the statistics, approximately a part of patients develop cardiac dysfunction at a cumulative DOX dose of 250 mg/m^2^ [37]. The mechanism of cardiotoxicity exerted by DOX is more complicated [38], to the extent that there is no effective clinical means of prevention or treatment. In our exploration of DOX‐induced cardiomyopathy, we have established a mouse model that successfully simulates the characteristics of cardiac atrophy, myocardial fibrosis, mitochondrial morphological disorder, atrial and ventricular enlargement, and obtained information from cardiac ultrasound examination similar to clinical symptoms (Figure 1). Simultaneously, we have discovered that DOX‐insulted H9c2 cardiomyocytes exhibited significant suppression in cell proliferation, excessive ROS and MDA, as well as increasing apoptosis (Figure S2, Figure 4). The nuclear localisation of FoxO3 has the ability to initiate the transcription of target genes that are involved in various cell biological functions, including regeneration and the response to oxidative stress [39, 40]. DOX, a ROS producer, is known to induce oxidative stress and cause damage in multiple cell types [29, 41, 42]. Mounting evidence suggests that increased oxidative stress attributes to the pathogenesis of DOX‐induced cardiotoxicity [43]. It has been previously demonstrated that *FoxO3* activates the expression of catalase in response to oxidative stress, thereby inhibiting cardiac hypertrophy [44]. Also, the deacetylation of *FoxO3* has been shown to enhance the expression of SOD2, thereby preventing the generation of ROS induced by ischaemia/reperfusion [45]. Conversely, *FoxO3* deficiency exacerbates heart injury caused by paraquat and inhibits oxidative stress through the upregulation of antioxidant enzymes, including SOD2 and CAT [28]. Our study further confirmed that DOX treatment stimulated the MDA and LDH accumulation, the accumulation of ROS in the mitochondria and reduced GSH, ATP and the expression of antioxidant genes, namely *SOD2* and *CAT* in heart (Figures 1 and 2). Moreover, our experiments using H9c2 cardiomyocytes demonstrated that the overexpression of *FoxO3* represses DOX‐induced ROS production (Figure 6). These findings speculate that *FoxO3* plays a central role in eliminating ROS against DOX‐induced cardiomyopathy.

Autophagy, a highly conserved intracellular degradation pathway, plays a crucial role in maintaining intracellular stability and is activated in response to myocardial damage associated with the heart [46, 47]. Previous studies have reported the detection of LC3II after injection of DOX, indicating an increase in autophagic vesicle synthesis and promotion of autophagy, possibly due to the cellular stress response to DOX [48]. Furthermore, Zhen Guo et al. reported the occurrence of autophagy in cardiac tissue of DOX‐induced cardiomyopathy patients and clarified useful markers predictive of the response to heart failure therapy [49]. We used the chronic DOX model (a multidose regimen) as a useful clinical reference in this study, where we observed that the degree level of autophagy in cardiomyocytes and heart was increased in Figures 3 and 4, as most patients receive long‐term treatment with DOX in clinical practice [50]. Additionally, we observed that DOX led to a significant accumulation of the number of both red and yellow puncta, which indicated that the DOX treatment accelerated the course of autophagic flux (Figure 6). Noteworthily, *FoxO3* has the ability to stimulate the transcription of autophagy initiator genes such as *ATGs*, *Beclin 1* and *ULK1* in the heart [24, 51]. Additionally, *FoxO3* influences gefitinib‐induced cardiotoxicity by promoting autophagy and upregulating LC3B [52]. Actually, the activation of heart‐specific expression of constitutive *FoxO3* is found to be sufficient to actuate autophagy [53]. *FoxO3* directly binds to the Mitofusin‐2 gene promoter, and inhibiting the FoxO3‐MFN2 axis reduced autophagosome accumulation [54]. Our research indicated that DOX‐induced oxidative stress stimulation promoted the phosphorylation of FoxO3, thereby activating autophagy (Figures 2, 3, 4 and Figure S2). Studies have shown that *FoxO3* knockdown suppresses autophagy [55], while we found that overexpression of *FoxO3* inhibited the phosphorylation of TSC2 and mTOR, thereby enhancing the expression of autophagic flux and autophagic proteins (Figures 5, 6, 7). Meanwhile, it has been reported that inhibition of autophagy initiation kinase ULK1 leads to the blocking of FoxO3‐TFEB‐mediated autophagy [56]. We found that activating *FoxO3* enhanced the protective effect of autophagy on cardiomyocytes and the heart by increasing the expression of LC3B and Beclin 1 (Figures 5 and 7). We also demonstrated that BafA1 inhibited autophagy, and overexpression of *FoxO3* relieved the inhibition of BafA1, thereby upregulating the LC3‐II/LC3‐I ratio, as well as strengthening the promotion of Rapa (Figure 5).

Our work demonstrated that DOX treatment enhanced the expression of autophagy marker *LC3B* and *Beclin 1*, and this effect was further intensified by the overexpression of *FoxO3* in H9c2 cardiomyocytes; however, it downregulated the p62 levels accordingly (Figure 5). The lipidated form of LC3‐I, known as LC3‐II, has been found to bind to autophagy and facilitate its maturation, making it an essential marker for the detection of autophagy [46]. This was supported by the observation that the expression of the active, membrane‐bound form of LC3B protein, LC3‐II, was increased both in vitro and in vivo, as well as boosting the formation of autophagosomes (Figure 6). Therefore, enhanced autophagy maintains cellular homeostasis and reduces the accumulation of reactive oxygen species, which is a crucial mechanism by which *FoxO3* protects against DOX cardiomyopathy by activating autophagy. Meanwhile, FoxO3 protein degradation was regulated by SIRT1 acetylation, leading to the transcriptional repression of FoxO3‐driven BNIP3 and blocking mitophagy initiation [57]. Restoring Sirt1 expression and activating FoxO3 could reduce cardiac injury and improve cardiac function in ischaemia/reperfusion models [58]. SIRT1 elevated FoxO3 expression through SIRT1‐mediated deacetylation of FoxO3 in the AngII treatment‐induced H9c2 cells hypertrophy model [59]. Moreover, SIRT1 is an NAD^+^‐dependent deacetylase that coordinates antioxidation, anti‐apoptosis and energy metabolism by modifying various transcription factors such as FoxO3 and NRF2 [60, 61, 62]. NRF2 is the main regulator of oxidative stress, and DOX inhibits NRF2 nuclear translocation, which leads to the alteration of antioxidant enzyme expression involved in SOD2 and CAT [63]. NRF2 is also involved in regulating mitochondrial biosynthesis, autophagic flow and anti‐inflammatory responses. The latest research has found that Cynaroside reduces the ROS level in the DOX myocardium by activating the AMPK/SIRT3/NRF2 pathway and significantly inhibits the apoptosis of cardiomyocytes, thereby improving cardiac function [64]. Furthermore, carvedilol potently mitigated cadmium‐induced cardiac intoxication by regulating KEAP‐1/Nrf2/HO‐1 and SIRT1/FoxO3 signals [65]. Therefore, transcription factor FoxO3, antioxidant‐inducible transcription factor NRF2, and deacetylase SIRT1 jointly regulate the ability of cardiomyocytes to cope with oxidative stress. Understanding the mechanism of action and interrelationship of these three in doxorubicin cardiomyopathy not only helps to clarify the nature of the disease but also provides molecular targets for the development of new cardiac protection strategies.

Besides, moderate autophagy induced by doxorubicin enhances protective effects. Studies have shown that Rapa‐activated autophagy decreased DOX‐induced cardiomyocyte death via inhibiting the apoptotic pathway [66]. Experiments have confirmed that the AMPK activator Astragalus polysaccharide can maintain energy homeostasis by enhancing autophagy flow and improving cell survival in animal models [67]. However, the physiological balance of autophagy is broken with the increase of doxorubicin dose or exposure time, and the overactivated autophagy programme may turn into apoptosis. The latest research has reported that excessive activation of mitochondrial autophagy leads to the accumulation of mtDNA released by damaged mitochondria in the cytoplasm, activating the cGAS‐STING pathway, triggering the inflammation and myocardial senescence [68]. Moreover, doxorubicin damages lysosomal function, resulting in the incomplete execution of the transcription factor EB (TFEB)‐driven autophagy programme, the obstruction of autophagosome‐lysosomal fusion, causing left ventricular atrophy and heart failure, and ultimately increasing mortality [69].

Several signalling pathways, including the mammalian target of rapamycinm mTOR pathway, have been mediated in autophagy regulation [70], with overactivated autophagy via mTOR in the presence of a cardiac remodelling model in vivo [71]. However, the relationship between FoxO3 and the mTOR signalling pathway in DOX‐induced cardiomyopathy remains unclear. Research has shown that mTORC1 inactivation phosphorylated the downstream kinase SGK1 and induced FoxO3 nuclear accumulation [72]. mTORC2 is the most important second‐layer kinase negatively regulating FoxO3 activity [73]. The reduction of the components of the mTORC1 and mTORC2 complexes leads to an increase in FoxO3 and downstream target genes that regulate autophagy and apoptosis [74]. In this study, we demonstrated that FoxO3 negatively regulated mTOR and promoted the procession of autophagy. In our study, we observed a downregulation of the phosphorylation of mTOR treated with DOX in Figures 3 and 4, further overexpression of *FoxO3* inhibiting the mTOR signalling pathway to activate autophagy levels (Figures 5 and 7). Considering that the constitutive activation of *FoxO3* in the hearts leads to reversible cardiac atrophy [53], the inactivation of mTOR, an important downstream target of *FoxO3*, can be interpreted as either a cytoprotective event or as an indication of cardiac atrophy. Our results are also consistent with the clinical search for therapeutic options for doxorubicin cardiomyopathy by exploring the autophagy phenomenon associated with mTOR targets [75]. In addition, our research demonstrates that *FoxO3* inhibits the phosphorylation of TSC2, thereby suppressing the activation of mTOR (Figure 7). Our findings clarified the point that exposure to DOX inhibited mTOR activity followed by autophagy upregulation in the heart and cardiomyocytes. Based on this study, we hypothesise that the downregulation of the phosphorylation of mTOR may be a beneficial effect mediated by *FoxO3*.

Moreover, ectopic expression of circ‐Foxo3 binding to senescence‐related proteins ID1 and E2F1, as well as stress‐related proteins HIF1α and FAK, was found to promote cellular senescence, while silencing circ‐Foxo3 decreased cell senescence [76]. Research has shown that Daidzein can effectively inhibit DOX‐induced heart failure in mice by suppressing fibrosis, cell apoptosis and oxidative stress, and regulating energy metabolism through the SIRT3/FoxO3 pathway [77]. Additionally, knockdown of *FoxO3* promoted doxorubicin‐induced cardiotoxicity in human cardiomyocytes [78]. Furthermore, recent research has demonstrated the selective modulation of *FoxO3* for therapeutic purposes, making it a potential therapeutic strategy for various clinical indications such as antihypertension, cardioprotection and antidiabetes agents [79, 80, 81]. Meanwhile, our recent research shows that *FoxO3* negatively controls cardiomyocyte proliferation and heart regeneration in postnatal mice [39]. Intrapericardial exosome therapy dampens cardiac injury via activating *FoxO3*, providing a promising candidate for the FoxO3 signalling pathway with an intrapericardial injection for cardiac repair [82]. Based on our research, overexpression of *FoxO3* enhances autophagy, inhibits oxidative stress and mTOR activation in the doxorubicin model; we propose that *FoxO3* initiates autophagy, resulting in a favourable outcome that mitigates the effects of DOX‐induced cardiomyopathy.

Notably, in this study, DOX exposure resulted in cardiac damage and dysfunction in mice manifesting as increased myocardium atrophy and fibrosis, increased levels of cardiac injury, whereas *FoxO3* intervention effectively improved these adverse effects induced by DOX. Hence, we demonstrated that the protective roles of *FoxO3* against DOX‐induced cardiotoxicity were ascribed to enhancing myocardial excessive autophagy, inhibiting excessive intracellular ROS and mTOR activity. In summary, our findings suggest that enhancing *FoxO3* can effectively alleviate doxorubicin‐induced cardiomyopathy by activating autophagy and indicate that *FoxO3* may represent a promising therapeutic target for the treatment of heart disease, particularly DOX‐induced cardiomyopathy in clinical practice.

In this study, the H9c2 cell line was treated with DOX to simulate a model of damaged cardiomyocytes. The H9c2 cell line offers technical benefits for examining cellular mechanisms. However, it lacks the electrophysiological characteristics of primary cells, such as spontaneous beating [83], and its proliferation characteristics are different from those of terminally differentiated mature human cardiomyocytes [84]. In addition, the metabolic characteristics of H9c2 cells, such as mitochondrial function [85], may not completely match the human pathological state. As a result, one should be careful when extending the cell line results to human DOX‐induced cardiomyopathy, and our findings should be interpreted accordingly.

## 5 Conclusions

The results of the present study demonstrate that DOX treatment increases the expression of phosphorylated FoxO3 in both heart and H9c2 cardiomyocytes, indicating that DOX causes upregulation of FoxO3 phosphorylation on Ser‐253. Besides, overexpression of *FoxO3* decreases DOX‐induced ROS levels, suggesting that *FoxO3* may have the potential mechanisms responsible for protection against DOX‐induced oxidative stress of the heart. Furthermore, our data also indicate that the cardioprotective effects of *FoxO3* against DOX‐induced cardiotoxicity in vitro and in vivo are associated with enhancing LC3B and Beclin 1 expression, leading to activation of autophagy, accumulation of autophagic flow and inhibition of mTOR activation. Considering the cardioprotective effects of *FoxO3*, we believe that it is worthy of additional molecular investigation in order to discover more effective and selective derivatives for use as the treatment of cardiomyopathy triggered by DOX.

## Author Contributions

**Zao‐Shang Chang:** data curation (equal), funding acquisition (lead), investigation (lead), methodology (equal), resources (lead), visualization (lead), writing – original draft (lead). **Le Wang:** conceptualization (equal), investigation (equal), software (equal). **Ju‐Xiang Zhou:** investigation (equal), resources (equal), software (equal). **Mei‐Xiu‐Li Li:** formal analysis (lead). **Meng‐Yun Yang:** software (equal). **Bin Luo:** resources (supporting). **Jia‐Jun Liu:** software (supporting). **Xiao‐Ye Sun:** resources (supporting). **Jing‐Bo Xia:** data curation (equal), funding acquisition (supporting), resources (equal), supervision (lead), visualization (supporting), writing – review and editing (lead).

## Ethics Statement

All experimental procedures conformed to the Guide for the Care and Use of Laboratory Animals (National Research Council, 8th Edition, 2011) published by the NIH. All animal protocols, including any relevant details, were approved by the ARRIVE (Animal Research: Reporting of In Vivo Experiments) guidelines and Institutional Animal Care and Use Committee (IACUC) of Guangzhou Sport University (Permit Number: 2021DWLL‐08).

## Conflicts of Interest

The authors declare no conflicts of interest.

## Supporting information


**Appendix S1:** Supporting Information.

**Appendix S2:** Supporting Information.

## Data Availability

The data that support the findings of this study are available from the corresponding author upon reasonable request.
