# Supplementary material for: FoxO3 Activation Alleviates Doxorubicin‐Induced Cardiomyopathy by Enhancing Autophagic Flux and Suppressing mTOR/ROS Signalling
Source: J Cell Mol Med. 2025 Aug 10;29(15):e70775. doi: 10.1111/jcmm.70775 (PMC12336054; doi:10.1111/jcmm.70775)
Supplement: Supplementary file 1 — Appendix S1: Supporting Information. [file JCMM-29-e70775-s001.docx]

**Materials and methods**

**Cell culture**

Cells were cultured as reported previously^1^. H9c2, rat embryonic heart tissue-derived cell line, were obtained from FuDan IBS Cell Center (FDCC, Shanghai, China), and cultured in high glucose DMEM (cat. no. 11965092, Gibco, USA) supplemented with 10 % fetal bovine serum (cat. no. A5256701, Gibco, USA), 1 % penicillin-streptomycin (100 U/ml penicillin and 100 µg/ml streptomycin) (cat. no. 15140122, Gibco, USA), at 37℃ in a humidified atmosphere with 5 % CO_2_. Culture medium was replaced every 2 to 3 days and cells were passaged when cell density reached a confluency of 90 % confluence.

In vitro experiment, H9c2 cardiomyocytes were cultured with 0.5 μM DOX (cat. no. 25316-40-9, MedChemExpress, USA) for 24 h, and the Control group was supplemented with PBS. In addition, H9c2 cells were treated with bafilomycin A1 (cat. no. 88899-55-2, BafA1, 10 nM; MedChemExpress, USA) or rapamycin (cat. no. 53123-88-9, RapA, 100 nM; MedChemExpress, USA) for 6 and 24 hrs respectively at 37℃ to block or promote autophagosome degradation, before treated with DOX for 24 h^2^.

**Overexpression plasmid construction**

The coding sequence (CDS) of *FoxO3* (NM_001376967, Supplementary Table 1 for the sequences) was amplified by KOD-Plus-Neo Kit (cat. no. KOD-401, Toyobo, Japan) and cloned into pcDNA3.1 expression vector as mentioned previously^3^. When cell density reached about 70%, H9c2 cells were transfected with plasmids at a final concentration of 2.5 μg/mL using Lipofectamine 3000 (cat. no. L3000150, Invitrogen, USA) in Opti-MEM reduced serum medium (cat. no. 31985070, Gibco, USA)^4^. The constructed overexpression plasmid pcDNA3.1-FoxO3 was further confirmed by DNA sequencing and Western blot.

**Determination of cell viability**

H9c2 cells were incubated with DOX (0, 0.5, 1, and 2 μM) for 24 h, followed by cell viability analysis using the CCK-8 Cell Counting Kit (cat. no. C0039, Beyotime Biotechnology, China) in accordance with the manufacturer’s instructions, as previously described^5^. Optical density values at 490 nm (OD490) were analyzed using a Multiscan Spectrum (Thermo Fisher Scientific, USA). Assays were performed in triplicate in three independent experiments. Cell viability % = (OD490 of each treated group/average OD490 of the blank Control group) × 100%.

**Determination of intracellular ROS**

The intracellular ROS generation was evaluated by measuring the oxidation of 5-(and 6)-chloromethyl-2’7’-dichlorodihydrofluorescein diacetate (CM-H_2_DCFDA, cat. no. C6827, ThermoFisher, USA) with flow cytometry according to the manufacturer’s instructions, as previously described^6^. Cells were detected with a flow cytometer (BD Biosciences, USA) at an excitation wavelength of 488 nm and an emission wavelength of 525 nm. Intracellular ROS concentration was determined by the mean fluorescence intensity (MFI) of 10,000 cells.

**Isolation of total mRNA and quantitative real-time PCR**

In brief^7^, total RNA was isolated using Trizol (cat. no. A33251, ThermoFisher, USA) from heart tissue and H9c2 cells according to the protocol of the manufacturer. Subsequently, cDNA was prepared using a cDNA reverse transcription kit (cat. no. 4368813, ThermoFisher, USA). Quantitative real-time PCR analysis was performed in SYBR qPCR Master Mix (cat. no. Q712-02, Vazyme Biotech, China). The sequences of primers for quantitative real-time PCR were listed in figure S1B and the primers were synthesized by Sangon Biotech, ShangHai, China. GAPDH was used as the internal standard Control to normalize gene, and fold changes in the expression of each gene were calculated by the comparative threshold cycle (Ct) method using the △△Ct method.

**Western blot**

According to our previous report^8^, H9c2 cells and heart tissue for SDS-PAGE were lysed in RIPA buffer (cat. no. P0013B, Beyotime Biotechnology, China) containing protease and phosphatase inhibitor (cat. no. PPC1010-1ML, Sigma, Germany). Protein concentration was determined using the BCA Protein Assay (cat. no. P0009, Beyotime Biotechnology, China). Proteins (30 μg) were separated in SDS-PAGE gels and then transferred to PVDF membranes. Concentrations used for each primary antibody are as follows: pFoxO3-Ser253 (cat. no. 9466S, CST, 1:1000 dilution), FoxO3 (cat. no. 12829S, CST, 1:1000 dilution), pmTOR-Ser2448 (cat. no. ab109268, Abcam, 1:1000 dilution), mTOR (cat. no. 2972S, CST, 1:1000 dilution), p62 (cat. no. A19700, Abclonal, 1:1000 dilution), Beclin 1 (cat. no. 66665-1-Ig, Proteintech, 1:1000 dilution), LC3B (cat. no. 14600-1-AP, Proteintech, 1:1000 dilution), Bax (cat. no. 2772S, CST, 1:1000 dilution), Bcl2 (cat. no. 15071S, CST, 1:1000 dilution), pTSC2-Thr1462 (cat. no. 3617S, CST, 1:1000 dilution), TSC2 (cat. no. 4308S, CST, 1:1000 dilution) and GAPDH (cat. no. 60004-1-Ig, Proteintech, 1:4000 dilution). Secondary antibodies used are following: goat-anti-mouse (cat. no. SA00001-1, Proteintech, 1:4000); goat-anti-rabbit (cat. no. SA00001-2, Proteintech, 1:2000). Signals were imaged with a gel imaging system (BIO-RAD, California, USA) and quantitative image analysis was performed using Image-Pro Plus version 6.0 software according to the manufacturer’s protocol.

**Autophagic flux assay (by GFP-mCherry-LC3B)**

According to the previous study^9^, in brief, H9c2 cells transfected with the GFP-mCherry-LC3 adenovirus (cat. no. C3011, Beyotime Biotechnology, China) for 24 h. Then cells were transfected with FoxO3 overexpression, and subjected to indicated treatment. Images were acquired using a confocal microscope (LSM 700, Zeiss, Germany). Autophagy flux was then assessed by counting of the GFP positive/mCherry positive puncta (yellow, autophagosomes) and GFP negative/mCherry positive puncta (red, autolysosomes) in cells. Autolysosomes and autophagosomes per cell were quantified from at least 30 cells per group in triplicate experiments.

**Transmission electron microscopy detection**

The hearts were fixed in 3% glutaraldehyde and prefixed with 1% osmium tetroxide, then tissues were rinsed with phosphoric acid rinse solution and were dehydrated in a series of acetone solutions. Subsequently tissues were embedded in Epon. Finally, the semithin sections were stained with methylene blue, while ultrathin sections were double-stained with uranyl acetate and lead citrate. Images were captured using transmission electron microscopy (Hitachi TEM system (HT7800))^10^.

**Measurement of biochemical index**

Heart oxidative stress markers such as malondialdehyde (MDA), and myocardial injury marker such as lactate dehydrogenase (LDH), glutathione (GSH) were measured using kits from Shanghai Beyotime Biotechnology (cat. no. S0131M, cat. no. P0395S and cat. no. S0052). The detailed assay procedures were performed according to the manufacturer’s instructions^11,12^.

**Animal administration**

All experimental procedures conformed to the Guide for the Care and Use of Laboratory Animals (National Research Council, 8th Edition, 2011) published by the NIH. All animal protocols, including any relevant details, were approved by the Institutional Animal Care and Use Committee (IACUC) of Guangzhou Sport University (Permit Number: 2021DWLL-08). C57BL/6J mice, male, aged 7–8 weeks and weighing 18-20 g, were sourced from the Guangdong Laboratory Animal Centre (Laboratory Animal Production License No:SCXK (Guangdong) 2022-0002; Laboratory Animal Qualification Certificate No.:44007200117320). Mice were housed under suitable temperature (23 °C) and lighting (12 h light/12 h dark cycle) with a relative humidity of 55–70% in Guangdong Provincial Key Laboratory of Physical Activity and Health Promotion. Six mice were housed per cage with free access to standard food and water.

Throughout the study, continuous monitoring of the mice was conducted to assess health status, behavioral changes, and any signs of distress. This included regular body weight measurements, evaluations of coat condition, checks for skin damage, and inspections for eye and nose discharge. Additionally, food and water intake were recorded, social behaviors were observed, and any instances of abnormal grunting, unnatural body posture, or excessive licking or scratching were documented.

Euthanasia was carried out when mice exhibited severe illness, irrecoverable health issues, significant behavioral abnormalities, or upon reaching the experimental endpoint. The euthanasia procedure involved administering an overdose of anesthetic to ensure a rapid and painless death.

Only male 8-9 weeks old C57BL/6J mice purchased from Guangdong Experimental Animal Center were randomized to receive an intraperitoneal (i.p.) injection of DOX (MCE, USA) at 5 mg/kg body weight since the animal model literature we referred used only male mice to exclude potential gender related effects^13,14^. Briefly, mice were injected once a week for 4 times, with a cumulative dose of 20 mg/kg. On the seventh day after the last DOX administration, mice were subjected to echocardiography and other correlation analysis. The Control animals were subjected to the same procedures as experimental animals and infused with PBS (Phosphate Buffered Saline, the same volume of infused DOX).

For heart collection, mice were anaesthetised using pentobarbital sodium (30 mg/kg body weight, intraperitoneal injection). With regards to euthanasia, the tail of the mice were pulled hard, and the thumb and forefinger of the other hand quickly pressed the mice's head forcibly, which pulled to dislocate cervical vertebra.

**Echocardiography**

Cardiac function was evaluated by echocardiography, as described previously^15^. In general, echocardiography was performed on mice using a Vinno 6 ultrasound system (VINNO, China). Mice were anesthetized with a 1.5-2% isoflurane flow, and their heart rates were stable within the range of 400-450 BPM for detection. Parasternal short-axis (M-mode) views of each animal were taken, and cardiac analysis package was used to analyze the data.

**Histological analysis**

Heart samples were prepared as previously described^15^, and sections were made and used for histochemical staining including haematoxylin and eosin (H&E, cat. no. PK10031, Proteintech, USA) and Masson’s trichrome (Masson, cat. no. C0189M, Beyotime Biotechnology, China). The relative myocardial collagenous fiber area were quantified using Image-Pro Plus version 6.0 software.

***FoxO3* gene delivery in vivo through adeno-associated virus (AAV)**

The coding sequence (CDS) of *FoxO3* (NM_001376967, Supplementary Table 1 for the sequences) was cloned into AAV serotype-9 expressing plasmid. AAV9 viruses were packaged and produced using the AAV Helper-Free System (DongBio.Co.Ltd, Shenzhen, China). For AAV9-FoxO3 delivery in vivo, 100 μL of AAV (titer of 1 × 10^12^ v.g./mL) was administered by a tail vein injection to mice for a total of 5 injections. AAV9-NC (virus packaged with empty plasmid) served as Control^3^. The schematic of AAV9 virus injection can be found in Supplementary Figure 3A.

**Statistical analysis**

Data analysis was performed using GraphPad Prism 9.0, and data were shown as mean ± SEM for the number of experiments indicated in the legends of the figures. Multiple comparisons were performed using one-way analysis of variance (ANOVA) followed by Bonferroni multiple comparison post hoc test. Comparisons between two groups were performed by using unpaired Student’s *t* test and *P* < 0.05 was considered a statistically significant difference.

1. Spallarossa P, Altieri P, Garibaldi S, et al. Matrix metalloproteinase-2 and -9 are induced differently by doxorubicin in H9c2 cells: The role of MAP kinases and NAD(P)H oxidase. *Cardiovasc Res*. Feb 15 2006;69(3):736-45. <https://doi.org10.1016/j.cardiores.2005.08.009>.

2. Sishi BJ, Loos B, van Rooyen J, Engelbrecht AM. Autophagy upregulation promotes survival and attenuates doxorubicin-induced cardiotoxicity. *Biochem Pharmacol*. Jan 1 2013;85(1):124-34. <https://doi.org10.1016/j.bcp.2012.10.005>.

3. Chang ZS, Xia JB, Wu HY, et al. Forkhead box O3 protects the heart against paraquat-induced aging-associated phenotypes by upregulating the expression of antioxidant enzymes. *Aging Cell*. Oct 2019;18(5):e12990. <https://doi.org10.1111/acel.12990>.

4. Duan Q, Yang W, Zhu X, et al. Deptor protects against myocardial ischemia-reperfusion injury by regulating the mTOR signaling and autophagy. *Cell Death Discov*. Dec 19 2024;10(1):508. <https://doi.org10.1038/s41420-024-02263-1>.

5. Zhou DC, Su YH, Jiang FQ, et al. CpG oligodeoxynucleotide preconditioning improves cardiac function after myocardial infarction via modulation of energy metabolism and angiogenesis. *J Cell Physiol*. May 2018;233(5):4245-4257. <https://doi.org10.1002/jcp.26243>.

6. Mustafa AM, Ashry R, Kramer OH. Monitoring Changes in Intracellular Reactive Oxygen Species Levels in Response to Histone Deacetylase Inhibitors. *Methods Mol Biol*. 2023;2589:337-344. <https://doi.org10.1007/978-1-0716-2788-4_22>.

7. Liang CQ, Zhou DC, Peng WT, et al. FoxO3 restricts liver regeneration by suppressing the proliferation of hepatocytes. *NPJ Regen Med*. Jun 24 2022;7(1):33. <https://doi.org10.1038/s41536-022-00227-6>.

8. Xia JB, Liu K, Lin XL, et al. FoxO3 controls cardiomyocyte proliferation and heart regeneration by regulating Sfrp2 expression in postnatal mice. *Nat Commun*. Mar 14 2025;16(1):2532. <https://doi.org10.1038/s41467-025-57962-9>.

9. Wei LX, Cui Y, Lv YL, et al. Lycium barbarum polysaccharide protects BMSCs against cadmium-induced suppression of osteogenic differentiation by modulating autophagy. *Ecotoxicol Environ Saf*. Apr 15 2025;295:118148. <https://doi.org10.1016/j.ecoenv.2025.118148>.

10. Zhang J, He Z, Fedorova J, et al. Alterations in mitochondrial dynamics with age-related Sirtuin1/Sirtuin3 deficiency impair cardiomyocyte contractility. *Aging Cell*. Jul 2021;20(7):e13419. <https://doi.org10.1111/acel.13419>.

11. Chen Q, Wang Y, Wang J, et al. Lipotoxicity Induces Cardiomyocyte Ferroptosis via Activating the STING Pathway. *Antioxid Redox Signal*. Feb 2025;42(4-6):184-198. <https://doi.org10.1089/ars.2023.0510>.

12. Liu WB, Wang SS, Zhang X, et al. Enhanced Cardiomyocyte NLRP3 Inflammasome-Mediated Pyroptosis Promotes d-Galactose-Induced Cardiac Aging. *J Am Heart Assoc*. Jul 16 2024;13(14):e032904. <https://doi.org10.1161/JAHA.123.032904>.

13. Mukhopadhyay P, Batkai S, Rajesh M, et al. Pharmacological inhibition of CB1 cannabinoid receptor protects against doxorubicin-induced cardiotoxicity. *J Am Coll Cardiol*. Aug 7 2007;50(6):528-36. <https://doi.org10.1016/j.jacc.2007.03.057>.

14. Neilan TG, Jassal DS, Perez-Sanz TM, et al. Tissue Doppler imaging predicts left ventricular dysfunction and mortality in a murine model of cardiac injury. *Eur Heart J*. Aug 2006;27(15):1868-75. <https://doi.org10.1093/eurheartj/ehl013>.

15. Lei Y, VanPortfliet JJ, Chen YF, et al. Cooperative sensing of mitochondrial DNA by ZBP1 and cGAS promotes cardiotoxicity. *Cell*. Jul 6 2023;186(14):3013-3032 e22. <https://doi.org10.1016/j.cell.2023.05.039>.
