# Supplementary material for: FoxO3 Activation Alleviates Doxorubicin‐Induced Cardiomyopathy by Enhancing Autophagic Flux and Suppressing mTOR/ROS Signalling
Source: J Cell Mol Med. 2025 Aug 10;29(15):e70775. doi: 10.1111/jcmm.70775 (PMC12336054; doi:10.1111/jcmm.70775)
Supplement: Supplementary file 2 — Appendix S2: Supporting Information. [file JCMM-29-e70775-s002.docx]

**Supplementary information**

**FoxO3 activation alleviates doxorubicin-induced cardiomyopathy by enhancing autophagic flux and suppressing mTOR/ROS signaling**

Zao-Shang Chang, Le Wang, Ju-Xiang Zhou, Mei-Xiu-Li Li, Meng-Yun Yang, Bin Luo, Jia-Jun Liu, Xiao-Ye Sun, Jing-Bo Xia

Supplementary Figure 1. Other related cardiac function indicators and primer sequences for analyzing gene expression.

Supplementary Figure 2. DOX administration inhibits the activation of *FoxO3* in H9c2 cardiomyocytes.

Supplementary Figure 3. Overexpression of *FoxO3* improves cardiac dysfunction in DOX-induced cardiomyopathy mice.

Supplementary Table 1. FoxO3 CDS sequence.


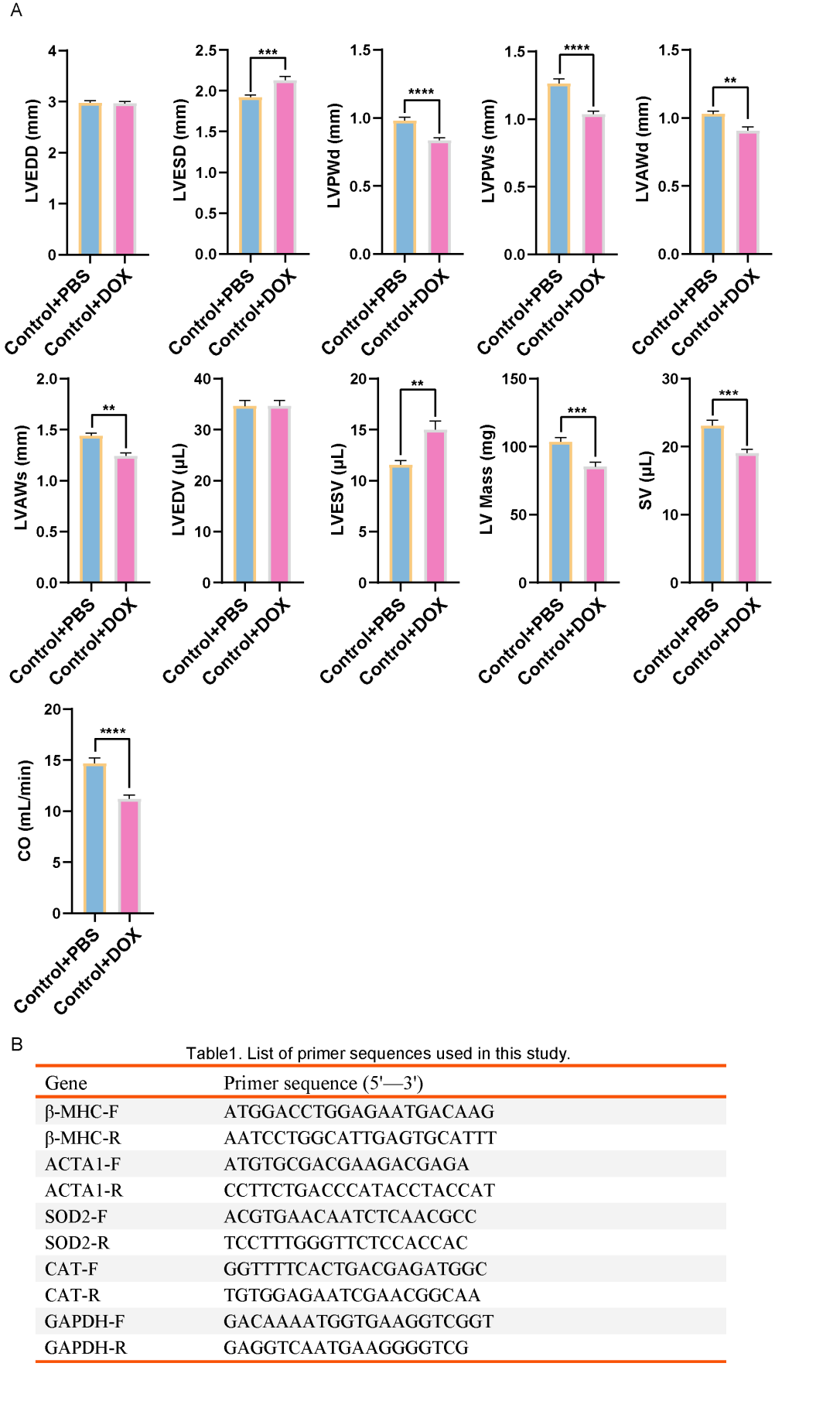


Supplementary Figure 1. Other related cardiac function indicators and primer sequences for analyzing gene expression. (A) Cardiac function indexes at the end of the experiment (n = 15). (B) Primer sequences were listed in this study. Data are presented as Mean ± SEM, ***p* < 0.01, ****p* < 0.001 and *****p* < 0.0001.


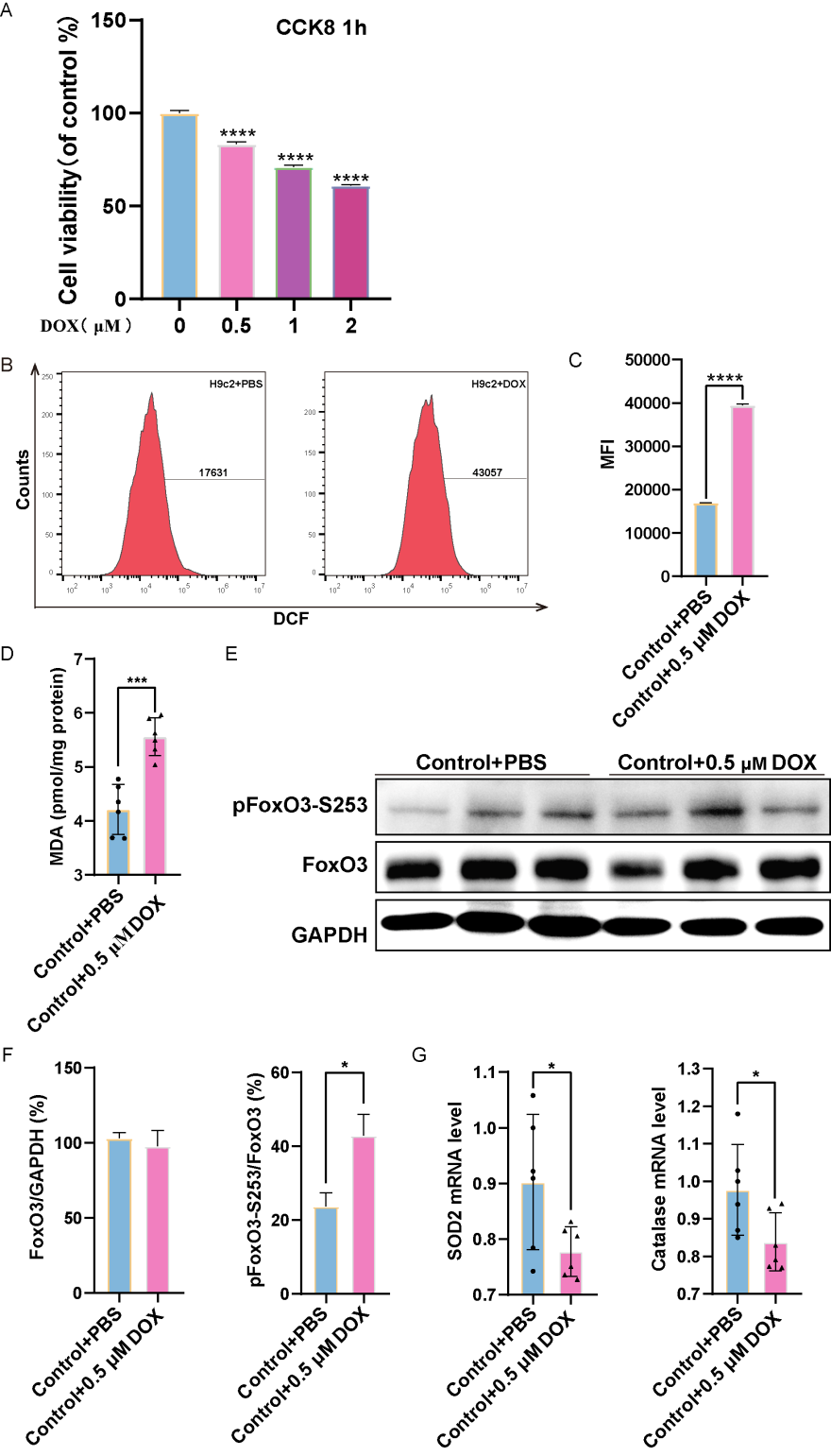


Supplementary Figure 2. DOX administration inhibits the activation of *FoxO3* in H9c2 cardiomyocytes. (A) The cell viability was determined by CCK8 assays at indicated time points after different incubation concentrations of DOX. (B) ROS detection by CM-H_2_DCFDA in flow cytometry. (C) Quantification of mean fluorescence intensity (MFI) (n = 3). (D) MDA in H9c2 cardiomyocytes was quantified (n = 6 per group). (E) Western blot images of FoxO3 and phospho-FoxO3-Ser253. (F) FoxO3 expression and phospho-FoxO3/FoxO3 ratio in H9c2 cardiomyocytes (n = 3). (G) *SOD2* and *CAT* mRNA expression in H9c2 cardiomyocytes. Data are presented as Mean ± SEM, **p* < 0.05, and *****p* < 0.0001.

**
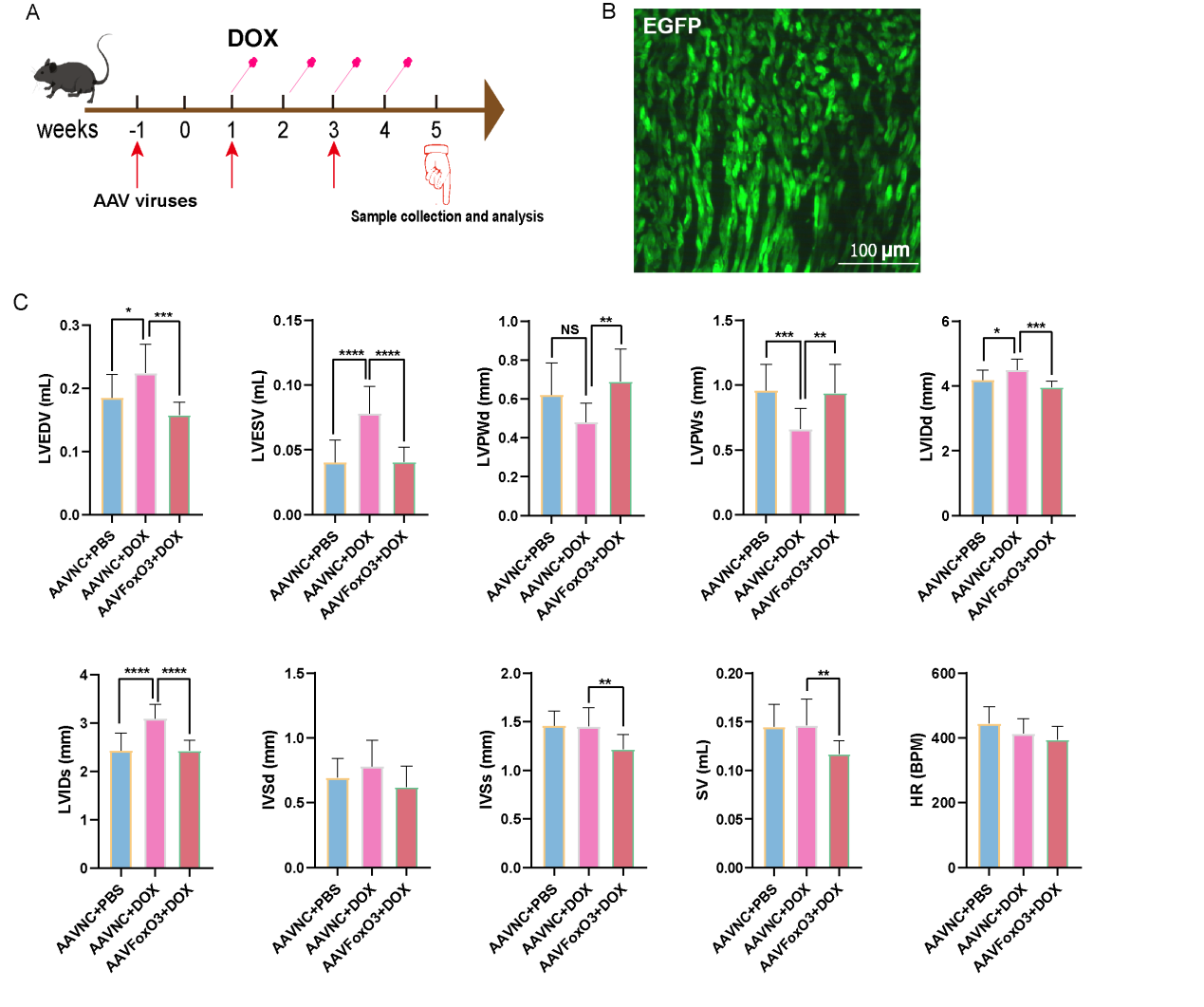
**

Supplementary Figure 3. Overexpression of *FoxO3* improves cardiac dysfunction in DOX-induced cardiomyopathy mice. (A) Schematic of AAV9 virus injection experiment designed to overexpress *FoxO3* in hearts exposing to DOX. The figure was drawn by Figdraw. (B) Representative images of AAV serotype-9 expressing plasmid reporter gene (EGFP) expression. (C) Cardiac function indexes at the end of the experiment (n = 20). Data are presented as Mean ± SEM, **p* < 0.05, ***p* < 0.01, ****p* < 0.001 and *****p* < 0.0001.

**Supplementary Table 1. FoxO3 CDS sequence**

| Mus musculus forkhead box O3 (FoxO3), transcript variant 1, mRNA |
| --- |
| NM_001376967 |
| atggcagaggcaccagcctccccggtcccgctctctccgctcgaagtggagctggacccagagttcgagccacagagtcggccacgctcctgtacgtggcccctgcagaggccggagctgcaggcgagcccggccaagccctcgggggagacggccgcagactccatgatccccgaggaggacgacgatgaagacgacgaggacggcggcggccgagccagctcggccatggtgatcggtggcggcgtgagcagcacgctgggttccgggctgctcctcgaggattcggccatgctgctggctccaggagggcaggacctcgggtcggggccagcgtccgccgcaggcgctctgagtgggggcacgccgacgcagctgcagcctcagcagccactgccacagccgcagccgggggcggctgggggctctgggcaaccaaggaaatgctcctcgcggcggaatgcctgggggaacctgtcctatgccgacctgatcacccgcgccatcgagagctccccggacaaacggctcactttgtcccagatctacgagtggatggtgcgctgtgtgccctacttcaaggataagggcgacagcaacagctctgcgggctggaagaactccatccggcacaacctgtccctgcacagccgcttcatgcgcgttcagaatgaaggcacgggcaagagctcttggtggatcatcaaccccgatgggggaaagagcgggaaggccccccggcggcgtgcggtctccatggacaacagcaacaagtacaccaagagccgaggccgggcagccaagaagaaggcggccctgcaggctgccccagagtcggcagacgacagtccttcccagctctccaagtggcctggcagccccacgtcccgcagcagcgacgagctggatgcgtggaccgacttccgctcgcgcaccaattccaacgccagcaccgtgagcggccgcctgtcgcccatcctggcaagcacggagctggatgacgtccaggatgatgatggacccctgtcccccatgctgtacagcagctctgccagcctgtcgccctccgtgagcaagccgtgtactgtggagcttccgcggctgacggacatggccggcaccatgaatctgaatgatgggctggccgagaacctcatggacgacctgctggataacatcgcgctcccgccatcgcagccatcgcctcctggcgggcttatgcagcggggctccagcttcccatataccgccaagagctccggcctgggctccccaaccggctccttcaacagtaccgtgtttggaccttcgtctctgaactccttgcgtcagtcacccatgcagactatccaggagaacagaccagccaccttctcttccgtgtcacactacggcaaccagacactccaagacctgcttgcttcagactcactcagccacagcgacgtcatgatgacccagtcggaccccttgatgtctcaggctagcaccgccgtgtccgcccagaatgcccgccggaacgtgatgcttcgcaacgatccaatgatgtcctttgctgcccagcctacccaggggagtttggtcaatcagaacttgctccaccaccagcaccaaacccagggcgctcttggtggcagccgtgccttgtcaaattctgtcagcaacatgggcttgagtgactccagcagccttggctcagccaaacaccagcagcagtctcccgccagccagtctatgcaaaccctctcggactctctctcaggctcctcactgtattcagctagtgcaaaccttcccgtcatgggccacgataagttccccagtgacttggacctggacatgttcaatgggagcttggaatgtgacatggagtccatcatccgtagtgaactcatggatgctgacgggttggattttaactttgactccctcatctccacacagaacgttgttggtttgaatgtggggaacttcactggtgctaagcaggcctcatctcaaagctgggtaccaggctga |
